# Impact of the observers' experience on daily prostate localization accuracy in ultrasound‐based IGRT with the Clarity platform

**DOI:** 10.1120/jacmp.v15i4.4795

**Published:** 2014-07-08

**Authors:** Christian Fiandra, Alessia Guarneri, Fernando Muñoz, Francesco Moretto, Andrea Riccardo Filippi, Mario Levis, Riccardo Ragona, Umberto Ricardi

**Affiliations:** ^1^ Department of Oncology Radiation Oncology Unit, University of Torino Turin Italy; ^2^ Radiation Oncology Department Azienda Ospedaliera Città della Salute e della Scienza Turin Italy

**Keywords:** IGRT, ultrasound, Clarity

## Abstract

The aim of this study is to evaluate the accuracy of daily prostate localization with ultrasound imaging of various radiation oncologists with nonhomogeneous expertise. For ten patients who underwent radical radiotherapy for localized prostate cancer, 11 radiation oncologists reviewed daily ultrasound scans acquired during three different treatment sessions. The average values of two senior radiation oncologists, considered to be expert observers, were selected as reference. The remaining nine observers were divided into two groups, Group 1 and Group 2, with more and less than one year of experience, respectively. The recorded shifts in prostate position were divided in three classes: <3 mm, 3–5 mm, and > 5 mm. Deviations from reference were less than 3 mm in all directions in 91% and 81% of measurements in Groups 1 and 2, respectively. The maximum difference in terms of root mean square error (RMSE) was reported for superior‐inferior (SI) direction, in particular a mean difference of 3.24 mm was observed for Group 2 in respect to the reference; moreover RMSE was 1 and 1.3 mm higher for Group 2 for anterior‐posterior (AP) and left‐right (LR) directions, respectively. The difference between Groups 1 and 2 was significant *(p* < 0.01) for all directions. The mean values for the shifts in all three directions between Group 1 and the references were 0.235 mm, 0.385 mm, and 0.009 mm for the LR, SI, and AP directions, respectively. The position of the prostate gland is more easily detectable (p=0.02) in the AP direction, while the visibility is lower for LR (p=0.02) and SI boundaries (*p* < 0.05). The observers' experience is essential for positioning the target correctly; therefore, a training period is recommended before putting the system into clinical practice.

PACS number: 87.63.dh

## I. INTRODUCTION

Image guidance nowadays represents an essential part of modern radiotherapy for prostate cancer. Direct imaging of the prostate gland and daily verification of its position during each treatment session is essential for reducing the margins and, consequently, avoiding the organs at risk such as rectum and bladder. Several image guidance methods have been investigated in recent years, including tumor tracking with intraprostatic fiducial markers, cone‐beam computed tomography (CBCT), and ultrasound‐based imaging (US‐image guidance).^1^, ^2^, ^3^, ^4^, ^5^, ^6^, ^7^, ^8^ Ultrasound‐based systems are volumetric and offer better soft‐tissue visualization compared to CBCT, without additional exposure to ionizing radiations.^(9)^ Several studies have shown that US‐based IGRT represents a reliable system for image guidance;^3^, ^7^, ^8^ various other technical solutions may be used for ultrasound IGRT in clinical practice, either based on intramodality or cross‐modality verification methods.^(3)^ Concerning other image guidance methods, optimal imaging may depend on the operators' experience.^10^, ^11^

The aim of this study is to evaluate the accuracy of daily prostate localization of various radiation oncologists with nonhomogeneous expertise in ultrasound imaging.

## II. MATERIALS AND METHODS

We have been testing the Clarity platform (Clarity System, Elekta, Stockholm, Sweden) in our hospital since 2008. It is a three‐dimensional target positioning device that allows intramodality verification by comparing the ultrasound images obtained before each treatment session, with the ultrasound images obtained at the time of CT‐simulation. The Clarity 3D ultrasound system consists of two US units (one located in the CT room, ${\text{Clarity}}_{\text{Sim}}$, and a second one in the treatment room, ${\text{Clarity}}_{\text{Guide}}$), and a special workstation for coregistration and image storage. An optical tracking system (OTS) is used for tracking the position and orientation of the 3D US probe. The OTS is registered to the laser systems in both the CT room and the treatment room.

After the planning CT scan has been carried out, a freehand axial sweep is acquired; the OTS detects an array of infrared reflectors affixed to the probe handle throughout the sweep. The sweep is then reconstructed to generate 3D ultrasound images.

A guidance structure in the workstation, defined as Positioning Reference Volume (PRV), is delineated on the ${\text{Clarity}}_{\text{Sim}}$ scan and used as reference on the ${\text{Clarity}}_{\text{Guide}}$ scan. During each treatment session a freehand axial sweep is acquired and then segmented in axial and sagittal slices. The daily PRV is then aligned with the reference PRV, first automatically by means of an optimization algorithm based on gray values and then manually according to the operator's experience. When the alignment is considered optimal, the system automatically accounts for the final target displacement by manually moving the couch in left‐right (LR), anterior‐posterior (AP), and superior‐inferior (SI) directions.

**Figure 1:**
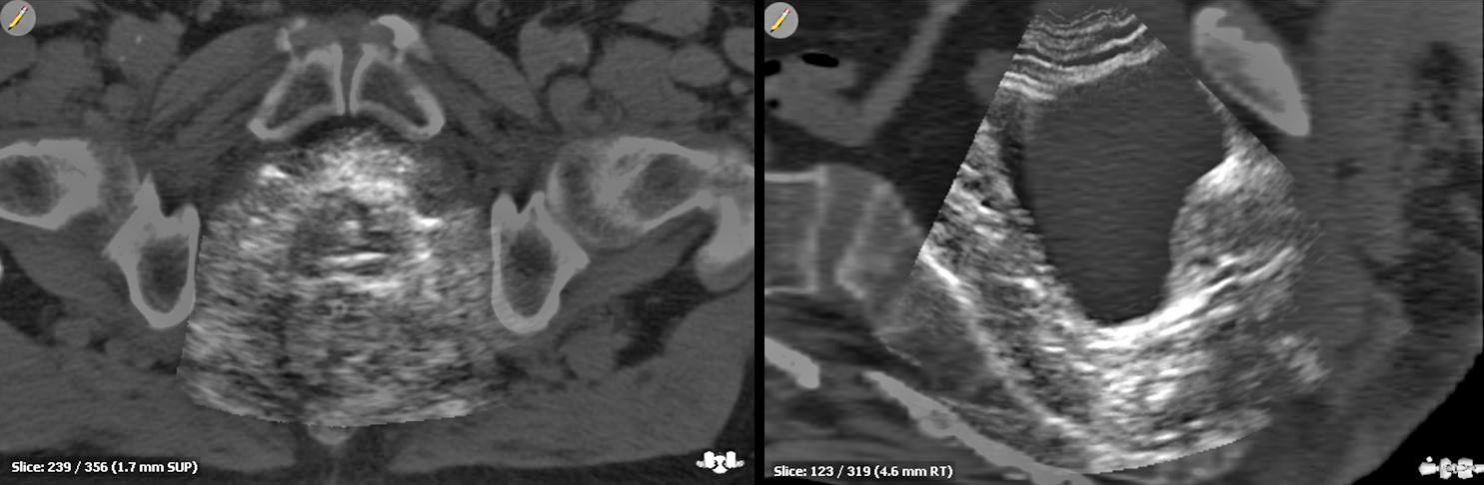
Typical US prostate visibility for patients enrolled for the study.

Ten patients undergoing radical radiotherapy for localized prostate cancer were selected for treatment with US IGRT. Each patient was deemed eligible for this procedure if visualization of prostate gland was considered acceptable by the two senior radiation oncologists. In general, about 10% of the patients were excluded due to unsatisfactory visibility (obesity, small gland volume or bad compliance of the patient to the diet or to our pretreatment protocol for filling the bladder). Eleven radiation oncologists reviewed daily ultrasound scans acquired during three different treatment sessions (fifth, tenth, and fifteenth fractions out of 26), and all shifts were recorded. The average value of two senior radiation oncologists, considered to be expert observers (more than five years of experience in US‐IGRT), were selected as reference for a correct prostate localization. A sample registration between US and its corresponding CT on sagittal and axial plane can be seen in Fig. 1.

The average value of prostate shifts obtained from two expert observers is considered “truth”, and the proximity of results from other nonexpert users to this value is then considered a measure of accuracy for these users. Consequently, the levels of accuracy and precision of the two expert observers were evaluated by analyzing the mean and standard deviation of the difference for each direction respectively.

The remaining nine observers were divided into two groups according to their experience in US IGRT: Group 1, including observers with more than 1 year of experience (number 1–5), and Group 2, with less than 1 year of experience (number 6–9). A basic statistical summary of the deviations between each observer compared with reference value, for each considered direction, was performed in terms of root mean square error (RMSE) and a statistical analysis between the two groups was carried out.

The relationship between the inaccuracy of shift determination and shift size was analyzed by means of standard deviation (SD) calculated for different classes of shift (<3 mm, 3–5 mm, and >5 mm). Then the relative frequency of shifts in prostate position measured by single observers in respect to the reference were divided into three classes and plotted to produce a frequency distribution for each class of shift for all directions. We then tested the difference of the shifts recorded in all three directions between the observers of Group 1 and the reference observers. The Student's *t*‐test was used to compare the results, with a p≤0.05 value for statistical significance.

## III. RESULTS

**Figure 2:**
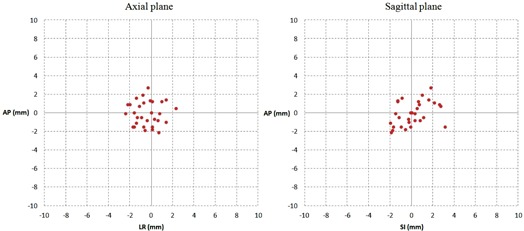
Scatter plot of points representing the values of the difference between the two reference observers respectively on the sagittal and axial plane.

Interobservers' variability was obtained by measuring the shifts between the various observers and the reference; Fig. 2 reports a scatter plot of the difference observed between the two reference observers plotted in both the sagittal and axial plane. The mean values of the difference between them were −0.4±1.2 mm,0.1±1.3 mm, and −0.1±1.4 mm, respectively, for LR, SI, and AP directions; more than 95% of the differences observed were within 3 mm. Table 1 illustrates the spread of shifts in the three directions for all observers in terms of RMSE. Total RMSE was reported for both groups for each direction. The maximum difference was reported for SI direction (3.24 mm for Group 2); for Group 2, RMSE was 1 and 1.3 mm higher for AP and LR direction, respectively, than Group 1. Significant differences between Group 1 and Group 2 were found for all considered directions (average values for Group 1: LR=1.32 mm,SI=1.69 mm,AP=2.05 mm; for Group 2: LR=2.67 mm,SI=3.24 mm,AP=3.09 mm).

**Figure 3:**
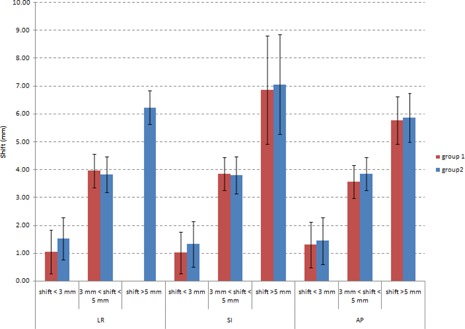
Histogram of mean deviations of each group analyzing three classes of shift; all different directions were analyzed and standard deviation for each bar is reported.

Figure 3 shows a histogram describing the mean values of the deviations of each group from reference observers. Values of 1.94 mm and 1.79 mm for standard deviations were observed for SI direction for Group 1 and Group 2, respectively; approximately 1 mm of standard deviation was found for remaining data.

**Figure 4:**
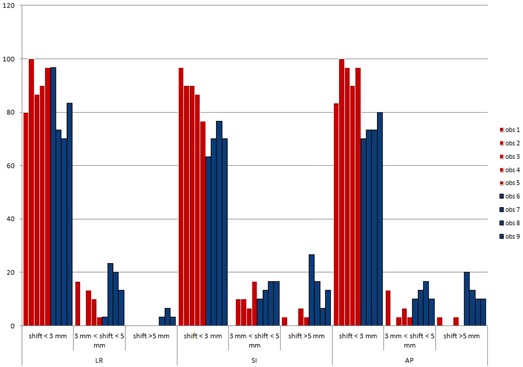
Histograms describing the frequency of shifts between each observer and the reference, divided by different directions (LR, SI, and AP). Red and blue bars represent, respectively, observers of Group 1 and Group 2.

The frequency distribution for every observer and group is presented in Fig. 4. Deviations from reference were less than 3 mm in all directions in 91% of measurements for Group 1 (range 80%–100%) and in 81% for Group 2 (range 70%–97%).

**Figure 5:**
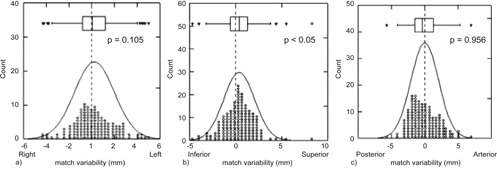
Distribution of the difference between Group 1 observers and reference observers in the three directions.

Figure 5 shows the distribution of all deviations of the operators of Group 1 compared to the expected zero value for the three directions considered. The smooth curve represents the normal distribution of deviation around its mean, which were 0.235 mm, 0.385 mm, and 0.009 mm for LR, SI, and AP direction, respectively. There was a significant difference (p<0.05) for the SI direction.

**Table 1:** RMSE of the difference between each observer and the reference for each direction; p‐value of Student's *t*‐test for each direction putting together all data for each group is reported in the last column

	*Observers Group 1*						*Observers Group 2*					
	*1*	*2*	*3*	*4*	*5*	RMS±SD *(all data Group 1)*	*6*	*7*	*8*	*9*	RMS±SD *(all data Group 2)*	^*p*^
LR (mm)	2.3	1.2	1.9	1.8	1.4	1.32±1.74	3.0	2.7	2.8	2.2	2.67±2.68	<0.01
SI (mm)	2.3	1.0	1.3	1.8	1.7	1.69±1.65	3.4	3.5	3.0	3.1	3.24±3.22	<0.01
AP (mm)	1.9	1.9	1.9	2.3	2.3	2.05±2.06	3.6	3.0	2.6	3.0	3.09±2.70	<0.01

## IV. DISCUSSION

The aim of the present study was to evaluate the extent of interobserver variability in correctly localizing the prostate gland by means of ultrasound imaging prior to each radiotherapy session, and its possible relationship with the experience of the single observers. Results show that the observers' experience is essential in correctly positioning the target, as the differences between Group 1 and Group 2 are 1 mm for AP direction, 1.5 mm for SI, and 1.3 mm for LR direction. Moreover, the differences are more pronounced in certain directions and this becomes evident when comparing Group 1 with the reference observers. The position of the prostate gland in AP direction appears to be well detected by all observers, and the orthogonal incidence between ultrasounds and the bladder and prostate wall (p=0.956; Fig. 4(c)) probably helps to visualize the prostate borders more clearly.

A significant difference between reference and Group 1 observers was reported in the SI direction (mean difference is 0.385 mm, p<0.05), which could be (at least partly) due to the worst image resolution along that direction.

Moreover, our data showed a good level of congruity between the two reference observers in localizing the prostate gland, as more than 95% of deviations were within 3 mm (Fig. 2). This 3 mm threshold can be considered as an indicator of the uncertainty of soft‐tissue visibility of the modality (i.e., an observer could be considered as expert if a certain percentage of measures are within this threshold).

Fuss et al.^(10)^ also defined 3 mm as being the intrinsic uncertainty threshold of operator interpretation of ultrasound images. Therefore, we applied the results of the present study in the clinical routine by choosing a cutoff in order to perform US‐IGRT independently — at least 80% of measurements should be within 3 mm in each direction, compared to the reference.

## V. CONCLUSIONS

Only radiation oncologists were involved in this study; however, for new operators a training period prior to the clinical use of the Clarity system is recommended in order to learn both the imaging and repositioning procedures.

## ACKNOWLEDGMENTS

The authors would like to thank Francesca Arcadipane, MD, Serena Badellino, MD, Sara Bouvet, MD, Jacopo Di Muzio, MD, Emanuela Pelle, MD, Cristina Piva, MD, Andrea Ruggeri, MD, and Elisabetta Trino, MD, for their participation in this study and for their help in data acquisition.
